# Patient and healthcare provider perspectives on adherence with antihypertensive medications: an exploratory qualitative study in Tanzania

**DOI:** 10.1186/s12913-021-06858-7

**Published:** 2021-08-18

**Authors:** Anbrasi Edward, Brady Campbell, Frank Manase, Lawrence J. Appel

**Affiliations:** 1 Department of International Health, Johns Hopkins University, Baltimore, USA; 2 University of Iowa Carver College of Medicine, Iowa City, USA; 3 Community Center for Preventive Medicine, Dar es Salaam, Tanzania; 4 Department of Medicine, Johns Hopkins University School of Medicine, Baltimore, USA; 5 Departments of Epidemiology and International Health, Johns Hopkins Bloomberg School of Public Health, and Johns Hopkins School of Nursing, Baltimore, MD USA

**Keywords:** Antihypertensive medication adherence, Qualitative study, Tanzania

## Abstract

**Background:**

Poor medication adherence is an extraordinarily common problem worldwide that contributes to inadequate control of many chronic diseases, including Hypertension (HT). Globally, less than 14% of the estimated 1.4 billion patients with HT achieve optimal control. A myriad of barriers, across patient, healthcare provider, and system levels, contributes to poor medication adherence. Few studies have explored the reasons for poor medication adherence in Tanzania and other African countries.

**Methods:**

A qualitative study applying grounded theory principles was conducted in the catchment area of two semi-urban clinics in Dar es Salaam, Tanzania, to determine the perceived barriers to HT medication adherence. Ten key informant interviews were conducted with healthcare providers who manage HT patients. Patients diagnosed with HT (SBP ≥ 140 and DBP ≥ 90), were randomly selected from patient registers, and nine focus group discussions were conducted with a total 34 patients. Inductive codes were developed separately for the two groups, prior to analyzing key thematic ideas with smaller sub-categories.

**Results:**

Affordability of antihypertensive medication and access to care emerged as the most important barriers. Fee subsidies for treatment and medication, along with health insurance, were mentioned as potential solutions to enhance access and adherence. Patient education and quality of physician counseling were mentioned by both providers and patients as major barriers to medication adherence, as most patients were unaware of their HT and often took medications only when symptomatic. Use of local herbal medicines was mentioned as an alternative to medications, as they were inexpensive, available, and culturally acceptable. Patient recommendations for improving adherence included community-based distribution of refills, SMS text reminders, and family support. Reliance on religious leaders over healthcare providers emerged as a potential means to promote adherence in some discussions.

**Conclusions:**

Effective management of hypertensive patients for medication adherence will require several context-specific measures. These include policy measures addressing financial access, with medication subsidies for the poor and accessible distribution systems for medication refill; physician measures to improve health provider counseling for patient centric care; and patient-level strategies with reminders for medication adherence in low resource settings.

**Supplementary Information:**

The online version contains supplementary material available at 10.1186/s12913-021-06858-7.

## Background

Hypertension (HT) is a leading risk factor for cardiovascular disease, affecting approximately 1.4 billion people worldwide [1]. Low-and-middle income countries (LMIC) have a disproportionate burden of HT, with only one fourth of those treated under control [2]. Awareness, treatment, and adherence to treatment for HT have been reported to be low for most African countries [3]. This is of special concern in Tanzania, where the estimated prevalence of HT is approximately 28%, with higher estimates in urban than rural areas [4, 5]. In Tanzania, only a third of patients who are diagnosed with HT seek care [6], with even fewer, only 3–23%, reporting appropriate medication adherence [4–7]. Importantly, Tanzania has notably higher prevalence, and lower awareness, treatment, and control compared to other developing countries, including India, China, Ethiopia, Pakistan, Ghana, Egypt, and others [8].

Approximately 75% of patients who are diagnosed with HT do not achieve optimum blood pressure control and experience associated complications due to poor medication adherence [2, 9]. Non-adherence to medications has shown to be a key factor contributing to uncontrolled HT [10].

In low-and-middle-income economies, effective implementation of HT services at the primary healthcare level is hindered by several health system, health provider, healthcare environment, and patient level factors [3, 11]. At the system level, inherent deficiencies impede effective HT management including chronic health workforce deficits, poor provider competencies, geographic access barriers, lack of essential medications, and the complexity of HT guidelines [12–15]. At the provider level, adherence is related to the quality of the provider-patient relationship, patient trust, communication styles, and patient centeredness of treatment decisions [2].

Inequities in patient access and affordability appear to be major contributors to poor adherence [16]. Other patient level factors that have shown to be strong predictors of adherence to antihypertensive medications include age, place of residence, income, educational levels, number of prescriptions, free medications, and comorbidities [17, 18]. Barriers to optimal patient compliance include inadequate knowledge of medication importance, perceived threat of the disease, and prioritization of other life events [19].

Studies on patient medication adherence have shown that improvements can be achieved by lowering medication costs, providing free medications [20–23], enhancing patient education [24], empowering patients to have higher self-efficacy and provider trust [25], and utilizing combination therapy to decrease side-effects and pill burden [26]. While quantitative studies are useful for defining problems, and assessing the prevalence of issues, qualitative studies, such as ours, are designed to identify putative barriers to adherence with medications for HT. In this study, we assessed potential barriers as reported by patients and healthcare providers in a semi-urban setting in Tanzania.

## Methods

This qualitative study was jointly conducted by Johns Hopkins University (Baltimore, MD), and the Community Center for Preventive Medicine (Dar-es-Salaam, Tanzania). The Community Center provides comprehensive health services with multi-disciplinary strategies to screen and manage non-communicable diseases, particularly HT, for a catchment area of 300,000 semi-urban residents. The Center has received wide recognition from the national and global development sectors for leading innovations in patient centered care, engaging over 200 volunteer physicians and nurses from the private and public sectors for HT screening and management. A recent study at this clinic examined the quality of patient screening and management for HT but did not address barriers to medication adherence [27].

The Focus Group Discussion (FGD) guide was designed through partnership with local physicians and translated into Swahili. The guide was structured on six key themes: 1) knowledge of HT, 2) role of antihypertensive medication, 3) perceived barriers to medication adherence, 4) strategies for improving adherence, 5) perspectives on traditional medicine and lifestyle changes, and 6) physician counseling. The Key informant interview (KII) guide was developed in English for healthcare providers. The KII guide focused on 5 themes: 1) healthcare delivery factors, 2) patient related factors, 3) medication factors, 4) role of religion, and 5) strategies for improving adherence. Ten healthcare providers, with English language proficiency, who managed HT patients, were selected from the Community Center for Preventive Medicine and Kinyerezi government clinic, also located in the catchment area, to participate in the KIIs.

Patients, 30 years and above, attending the Community Center for Preventive Medicine and Kinyerezi clinic, and patients of the providers selected for the KII, were selected for the FGD. The study cohort was identified using purposeful sampling to identify hypertensive patients (clinical diagnosis of HT based on national guidelines (SBP > 140 and/or DBP >90mmhg on multiple visits), from the patient register. Subsequently, using systematic random sampling, male and female patients were selected to participate in the FGD. Selected patients were contacted during clinic visits or by phone to participate in the FGDs. Patients who were known to have hearing or mental health impairments were excluded. Four FGDs were conducted with female participants and five with male participants, during a two-week period in January 2020 using standard procedures [28].

Participants for the FGD were informed about the venue, time, and duration of the FGD, a few days prior to the event; despite their intent to participate, 2–5 of the participants were unable to join each FGD, although none declined participation. Since participants were selected from the patient register, the general profile was obtained from the records, but not documented for individual FGD participants. Other patient characteristics were not included in the patient profile for the FGDs. This decision was made to comply with the designation of ‘Exempt Human Subjects Research’ by the Johns Hopkins IRB. Although the ideal recommended sample for FGD is between 8 and 10 participants, mini FGDs with 3–6 members have been used to gain a better understanding of participant experiences for in-depth insights [29]. If participants have intense experiences and recommendations to share, it is considered a better strategy to have smaller number of participants for the FGDs. Based on the criteria of obtaining in-depth understanding about patient adherence, complexity of the content, number of themes, and management of patient dynamics, the sample in each FGD was likely sufficient [29–31].

In accordance with standard guidelines, we continued the FGDs, until no new themes emerged based on the research coordinators assessment. A total of 34 participants took part in the FGDs, with an average of 4 participants per FGD. The FGDs were planned for about 60–70 min. The focus groups were conducted by a facilitator who was a native Swahili speaker, and healthcare supervisor with previous qualitative research experience, particularly in conducting FGDs. The note taker was a bilingual Tanzanian native with a background in healthcare social work. The focus groups were supervised by a medical doctor, with public health experience. These recordings were kept on a secure device until they were transcribed. The discussions were conducted in close proximity to the clinics in locations that were accessible and familiar to the FGD participants, ensuring privacy and non-interruption.

The KIIs were conducted in the clinics in a private setting, to minimize disruption of the healthcare providers’ daily activities. A sample size of 10–12 KIIs has been shown to provide data saturation, with key thematic ideas being repeated [32]. Interviews were scheduled to last between 30 and 45 min and were conducted in English by one of the study team members, a co-author, who was a medical student also training for a Masters’ Program in Public Health. These interviews were also audio-recorded on a secure device, until they were transcribed. Monetary compensation was not provided to the patients or the physicians.

To ensure study robustness, we ensured appropriate selection of the research team, instituted a system for coding discrepancies, and ensured credibility and authenticity of the research process and thematic ideas investigated [33]. Inductive codes were developed for the various themes of the FGDs and KIIs, using standard procedures of grounded theory followed by deductive codes similar to other studies [34, 35]. The FGD audio record was transcribed by a native Swahili speaker and translated to English by a bilingual translator from the study team. Deductive codes were determined through collaboration of the co-authors, including a Tanzanian physician. The KIIs were transcribed, coded, and analyzed. Coding was completed in a systematic manner, with iterative review, by a research team member for both KIIs and FGDs, with consultation of a second member on any arbitrary statements. Data were then analyzed, using an inductive approach noting the frequency of key theme identification. While the FGD and KII were conducted during the same study period, there was no comparative approach between groups during the interview process, as they were conducted individually.

As the study was designed as an exploratory research in a specific semi-urban location, it did not include all the elements, but followed the general principles of a grounded theory approach [35, 36]. The themes were determined based on previous work from Nigeria and information from clinicians during the planning phase for a previous study on healthcare provider knowledge on HT [27, 34]. Traditional Grounded theory generates a conceptual theory that accounts for a pattern of behavior that is relevant to the research, however, there are factors that may distinguish differences between the approaches and the philosophical position of the researcher in the use of literature and approach to coding and analysis [35]. We applied several elements, selecting the participants with the specific condition and on antihypertensive medications, creating discussion guides and initial and intermediate coding; however, the data were too sparse to consider extensive interconnectedness, though a few themes that emerged in the focus groups were interconnected, like geographical access to clinics and affordability of care. While specific themes are not always associated with grounded theory, we used the framework to assist the analysis of the interview transcripts, applying the steps for the coding procedures. Briefly, this inductive method began with the assignment of a series of open codes to the transcripts of individual interviews, which were then grouped into clusters (concepts). From these concepts, broader categories and subcategories were generated, through a process of constant comparison or verification.

The EMERGE guidelines provide a structured methodology for adherence measures to address elements related to medication initiation, implementation, and discontinuation [37]. Due to structural differences, the full framework of the EMERGE guidelines were not considered. However, we included the phase of adherence, results, and operational definition at the persistence level, and utilizing measurement with self-reported data.

Ethical approval was obtained from the National Institute for Medical Research in Tanzania by the Community Center for Preventive Medicine, and from the Johns Hopkins Bloomberg School of Public Health Institutional Review Board, which considered the study as exempt human subject research. Informed consent was obtained from the health providers who participated in the KIIs and from the patients who participated in the focus group discussions.

## Results

FGD participants included 17 female and 17 male participants between the ages of 30–65. They were mostly farmers, housewives, and daily laborers from semi-urban areas. Only a few had completed formal secondary education, and most had never worked in the formal job sector.

### Patient reported barriers to medication adherence

Participant responses of barriers to antihypertensive medications were categorized into five major themes, with access to care and cost cited most commonly (Table 1).

**Table 1:** Patient Reported Barriers to Antihypertensive Medication Adherence from FGD (*n* = 34)

Category	Sub-Category	Concepts
1. Access Factors	Travel distance to healthcare facility Medication availability Lack of screening programs Physician Access	While pharmacies are more accessible, they are far more expensive than clinics (*n* = 8) There are extensive distances to travel to the clinic (*n* = 4) Hospitals are often out of stock (*n* = 3) Pharmacies will not sell shorter doses to decrease price (*n* = 1) There is no culture of screening, so people do not go (*n* = 9) There are no screening programs in place (*n* = 8) There are many barriers to even being seen by a physician, including a lack of specialists (*n* = 4) There is a shortage of physicians (*n* = 3)
2. Cost Factors	Affordability of medications High cost of healthcare screening	Medications are too expensive to buy (*n* = 25) No reduced cost for senior citizens as cited in the national health insurance schemes (*n* = 9) Taxes make medications more expensive (*n* = 2) People cannot be screened or properly check their BP due to high cost (*n* = 6)
3. Physician Factors	Poor counseling Lack of time to provide patient centered care Physician knowledge	Need sufficient counseling on prevention (n = 1) Do not inform patients how long they will be treated (*n* = 1) Physicians are distracted and not patient focused (*n* = 5) There is insufficient time for physicians to counsel (*n* = 3) Physicians are not educated enough on hypertension (*n* = 2)
4. Patient Factors	Lack of education and understanding about disease Stop taking the medicine Forgetting to take medicines Lifestyle Habits Role of religion	Patients do not have an understanding of hypertension (*n* = 15) Actively choose not to take medicines (*n* = 5) Patients do not even know they have hypertension (*n* = 3) Patients do not know severity of their disease (*n* = 3) Patients stop taking medicine once they feel better (*n* = 8) Patients get tired of taking medicine, so they will only occasionally take it or not take it at all (*n* = 5) Without seeing improvement, they stop medications (*n* = 2) Accidently forget to take medicines (*n* = 3) Elderly populations forget to take medicines (*n* = 3) Healthy food does not taste as good “Choose a less healthy lifestyle due to convenience or taste” (*n* = 6) People do not know how to maintain a healthy lifestyle (*n* = 2) It is very difficult to follow lifestyle advice (*n* = 3) The disease was sent by God (*n* = 1) Believe in letting God cure them (*n* = 3)
5. Medication Factors	Side Effects Herbal Medicines Prescription complexity	Patients are scared of side effects (*n* = 3) Misconceptions about side effects (*n* = 2) Side effects are too severe to continue taking medicine (*n* = 5) Utilize herbal medicines as replacements (*n* = 10) Polypharmacy is difficult for patients (*n* = 2)

Access barriers were commonly mentioned by the FGD participants. These included geographic access to health facilities and transport, access to information on HT, and access to HT screening and medications. Lack of routine blood pressure screening was also raised by the participants. It was explained that – “*In most cases, it is not until people go to the hospital or get sick, then they get to know that they are hypertensive, otherwise it hard to know, because we do not have a culture of doing health screening regularly” (FGD 7, P1).*

Cost was another major barrier, as the affordability of medications and health screening are both financially difficult for patients. A middle-aged female illustrated her perspectives on the issue of costs “*Everyone wants treatment, everyone wants good health, however not everyone can afford it” (FGD 8, P1)*. The perception and/or experience that medications were too expensive emerged in all FGDs, with 25 of 34 respondents identifying it as a major cause for poor medication adherence. A related issue raised by some participants was the lack of subsidized price schemes for senior citizens, despite having health insurance which is expected to cover medications (*n* = 9). Finally, participants expressed concern that “*people do not check their blood pressure due to the high cost they would incur” (FGD 2, P1),* indicating that cost is a barrier for screening and preventative care as well (*n* = 6).

The lack of knowledge about the importance of taking medications regularly was another barrier that emerged from the FGD. Many patients stopped medications when they felt better or their symptoms disappeared (*n* = 8), stating “*If I feel okay, I don’t take [medicine], rather I hide them. When I am not okay, I take them, then if the condition stabilizes, I hide them again” (FGD 4, P3).* One elderly female participant elaborated “*HT is not like other disease, in most cases we know less about it” (FGD 1, P6).* The cultural shifts, from traditional healthier food to a modernized culture of fried food and increased consumption of food with high salt was also cited (*n* = 6).

Physician related factors were also evident. The length and quality of the counseling provided were both barriers (*n* = 3), as they were not customized to the patient’s needs and understanding. Concerns about the healthcare provider being distracted and not patient-centered were also present (*n* = 5). This is especially concerning at the point of initial diagnosis; an elderly woman explained “*when I was diagnosed with HT, I didn’t get ample time to sit with the Doctor to explain to me about it” (FGD 4, P1)*.

The preference for and faith in the effectiveness of herbal and local medicines was commonly cited (*n* = 10). Herbal medicines are not a barrier if used as a supplement to physician prescribed medications; however, they are often used as a replacement for prescribed medications, as they are affordable, accessible, and acceptable within the local culture. The perception that herbal medicines also do not require frequent hospital visits was illustrated by this quote: “*when we use these traditional medicines we have faith in them that they cure, so we leave it that way, but we don’t screen to monitor the progress as we do in the hospitals” (FGD 1, P6).* Side effects (*n* = 5) and prescription complexity (*n* = 2) of physician prescribed medicines were also mentioned.

### Patient reported facilitators to antihypertensive medication adherence

Table 2 displays key themes that emerged from the FGD on facilitators of medication adherence .

**Table 2:** Patient Reported Facilitators for Antihypertensive Medication Adherence from FGD (n = 34)

Category	Sub-Category	Concepts
1. Access Factors	Travel distance to healthcare facility Insurance Factors Physician access Screening programs	Employ community health workers to increase access to care and adherence (*n* = 1) Education should be based in community outreach (*n* = 2) Increase insurance access to obtain free medicines (*n* = 2) Need to educate patients to come to clinic sooner (*n* = 2) Should screen all patients at clinic (*n* = 6) Utilizing public health screening campaigns (*n* = 3)
2. Cost Factors	Affordability of medications	Medications should be made free or subsidized (*n* = 11) Need to increase awareness and utilization of insurance (*n* = 2) Medications should be tax-exempt (*n* = 3)
3. Physician Factors	Proper counseling	Continue medication until told to stop by physician (*n* = 12) Provide counseling at every healthcare interaction (*n* = 3) Provide counseling about lifestyle changes (*n* = 10) Advise regular check-ups (*n* = 2) Spend ample time counseling in clinics (*n* = 2) Provide further patient education on prevention (*n* = 3) Counsel about harm of not adhering to medicines (*n* = 1)
4. Patient Factors	Importance of lifestyle changes Utilizing reminder systems	Utilizing exercises to be healthy (*n* = 6) Maintaining healthier dietary habits (*n* = 13) Reducing stress (*n* = 2) Receiving SMS text message reminders to take medication would help (*n* = 17) Employing memory cues around the house (*n* = 5) Setting alarms (*n* = 2) Always keeping medications on hand (*n* = 4) Utilizing family members to help with adherence (*n* = 6) Using public health campaigns to increase awareness (*n* = 4) Utilizing community support groups (*n* = 1) Require patients to come to clinic to take medicines (*n* = 1)
5. Medication Factors	Prescription complexity	Take medicine at same time every day (build routine) (*n* = 5)

Increasing access was the first facilitator mentioned, which included utilizing community health workers to address geographical barriers, awareness, and medication refills, along with health insurance to offset clinic and medication costs. Participants expressed the importance of early and continued screening for early detection of HT (*n* = 2). HT awareness campaigns were mentioned as a strategy to improve knowledge and awareness of HT and the importance of regular medication adherence. As one female participant noted, “*they should not wait for us to visit the health centers, they should organize public health education on prevention of HT” (FGD 4, P1).*

Offsetting the cost of antihypertensive medications was identified as being very important. Participants suggested that free or subsidized medication could be provided, similar to HIV/AIDS and TB programs (*n* = 11). This would enable patients to improve medication adherence and utilization of healthcare facilities *“Put simply* – *free is better (FGD 6, All participants)*. Another suggestion was tax exemption for medication “*if the taxes are waived, even in the low-income earners, they would be able to buy these medicines (FDG 9, P2)”.*

Certain physician factors were identified, including improving the quality of counseling, which would lead to better care for hypertensive patients. Participants believed that “*the Doctor should highly emphasize patient adherence (FGD 7, P2”).* Importantly, 12 of 34 participants indicated that physicians should emphasize the importance of continuing medicines until the physician says to stop. The significance of prevention was also discussed. Patients felt that if physicians could improve education, there would be less disease, explaining “*when you talk about prevention, we are talking about education. When you educate somebody, you prevent the disease (FGD 6, P2)”.*

Improving lifestyles and utilizing reminder systems to increase medication adherence were two sub-categories identified within patient factors. Counseling about healthy dietary habits (*n* = 13) and increasing the amount of exercise (*n* = 6), were mentioned most often by the participants. While many reminder systems were identified, the idea of using SMS text messaging was the most common (*n* = 17), because “*if you forget to use medicine, you are being reminded, and it can help”, and* “*The phone is always with us.” (FGD 8, P1).* Another suggested mechanism is to *“put a reminder alarm in my phone for drug taking time” (FGD 9, P1).* Participants also considered utilizing family members to help keep them accountable (*n* = 6).

Finally, medication factors to help adherence included creating a routine for taking prescriptions at the same time every day (*n* = 5), which is especially helpful for complex prescriptions. An elderly male explained “*when you wake up, you have to take medicine, otherwise you will forget” (FGD 1, P6).*

### Healthcare provider key informant interviews

A total of ten healthcare providers -- three nurse midwives and seven physicians -- who routinely screen and manage hypertensive patients participated in the KIIs. Four major coding categories were determined for the KIIs; healthcare related factors, patient related factors, medication factors, and religion (Table 3).

**Table 3:** Healthcare Provider Perceived Barriers for Patient Antihypertensive Medication Adherence from KII (*n* = 10)

Category	Sub-Category	Concepts
1. Healthcare Related Factors	Counseling Physician Training Physician Access Healthcare Costs Screening Access	Need more physician education surrounding hypertension diagnosis and medications (*n* = 2) Takes too much time to counsel (*n* = 1) No official hypertension training (*n* = 6) Lack of follow-up care (*n* = 1) Infrastructure is lacking, especially in rural areas (*n* = 2) Screening is too expensive (*n* = 1) Not enough patients diagnosed (*n* = 2)
2. Patient Related Factors	Adherence Lifestyle Factors Disease Knowledge Refusal	Forget to take medication (*n* = 1) Busy lifestyle causes people to forget (*n* = 5) Unwilling to change lifestyle (*n* = 5) Do not realize they are sick (*n* = 6) Do not understand the disease (*n* = 7) Unaware of long-term complications (*n* = 1) Unable to read education materials (*n* = 3) Refuse to take medications (*n* = 4)
3. Medication Factors	Side Effects Affordability	Side effects feel harmful (*n* = 2) Concern about long-term effects (*n* = 2) Pill Burden (*n* = 5) Medications are too expensive (*n* = 9)
4. Religion	Medication use discouraged Higher trust in religious leaders	Pastors advise to stop taking medicines (*n* = 2) Belief that traditional medicine is better (*n* = 4) Trust in traditional medicine leads to false belief in cure (*n* = 5)

### Healthcare provider perspectives on patient barriers to antihypertensive care

Healthcare related factors included aspects related to the lack of adequate training on patient counseling (*n* = 2), time required for patient counseling (*n* = 1), and lack of infrastructure in rural settings (n = 2). The most frequently mentioned barrier was the lack of adequate training (*n* = 6), “*physicians need more training on HT” (KII 7, physician)*.

Patient related factors were rooted in patients’ lack of understanding about the long-term complications of HT (*n* = 7), which was represented by other barriers including the unhealthy lifestyle of those engaged in a business lifestyle (*n* = 5), reluctance to change lifestyle (n = 5), or patients not understanding that HT is a condition that requires medicines (*n* = 6). Forgetfulness was also mentioned, along with reluctance for continued lifelong medication. A female physician (KII 8) explained this concept: “*When you ask patients if they have disease, they don’t know, they just know that providers told them to take medications”*. Another physician observed “*patients don’t want to take medications every day, and they are afraid to start them, because they do not want to continue them throughout their life” (KII 3, physician).*

Patient financial insecurity and inability to pay for medicines were addressed. One physician shared the following statement from one of his patients: “*Doctor, I don’t have any money to take my medicines” (KII 5, physician).* Another physician explained “*If you have $1 USD per day then it is impossible to afford [HT] medication” (KII 9, physician).* The possibility of experiencing harmful side effects or long-term effects of medications (*n* = 2) and pill burden (*n* = 5) were also commonly reported as medication related factors for patient non-adherence.

It was apparent that the church leaders played a critical role based on physician perceptions: “*pastors will say this disease is not part of your life, which influences patient’s perspectives and lead to non-adherence of medication” (KII 5, physician).* Healthcare providers also mentioned that patients stop taking medications if they felt that HT could be controlled by prayer (*n* = 2), and because they felt that traditional medicine was far more effective for treating HT (*n* = 4).

### Healthcare provider perspectives on facilitating factors for antihypertensive care

The categories for facilitating factors (Table 4) were similar to the barriers to care previously described. Healthcare providers made several recommendations for enhancing patient adherence to antihypertensive care which were related to the health system. Sub-categories included increased counseling, HT prevention efforts, public health campaigns, and physician training. The healthcare providers gave several suggestions, including increased clinic visits to provide counseling on lifestyle changes (*n* = 5), counseling for family members (*n* = 2), and individualized treatment plans: “*physicians need to work with patients to find programs that work for them to take medications” (KII 10, physician).* These were to be complemented by improving prevention efforts, i.e., screening all patients at the clinic (*n* = 3), increasing public health programs and campaigns (*n* = 4), building community coalitions (n = 2), engaging community health workers, and providing more training opportunities (n = 4). Examples of these already implemented solutions include “*a WhatsApp group focused on health education and exercise” (KII 3, physician),* and campaign “*through mass media; this is the only way we can raise awareness to the whole population” (KII 9, physician)*.

**Table 4:** Healthcare Provider Perceived Facilitators for Patient Antihypertensive Medication Adherence from KII (*n* = 10)

Category	Sub-Category	Concepts
1. Healthcare Related Factors	Counseling Prevention Efforts Public Health Campaigns Physician Trainings	Increase the number of clinic visits (*n* = 2) Provide education about lifestyle changes (*n* = 5) Counsel family members (*n* = 2) Education about long-term outcomes (*n* = 3) Emphasize prevention over treatment (*n* = 3) Screen every patient at health clinics (*n* = 3) Increase public health messaging (*n* = 4) Establish public health programming (*n* = 3) Build community coalitions (*n* = 2) Utilize community health worker (*n* = 2) More training opportunities (*n* = 4)
2. Patient Related Factors	Support Tools Insurance	Utilize younger family members (*n* = 3) Incorporate medicines into daily routine (*n* = 3) SMS text message reminders for medicines (*n* = 5) SMS reminders for clinic appointment visits (*n* = 3) Utilizing insurance (*n* = 3)
3. Medication Factors	Affordability Patient Self-Efficacy	Lower prices (*n* = 6) Medication specific counseling (*n* = 2)
4. Religion	Build Partnerships	Utilize religious leaders to share information (*n* = 4)

Suggestions for addressing patient related factors included the engagement of younger family members to remind older patients (*n* = 3), taking medications during a daily routine task (*n* = 3), SMS text messaging for clinic visit reminders (*n* = 3) and medication reminders (*n* = 5), and increasing coverage through health insurance schemes (*n* = 3). Technology strategies through SMS text messages was the most commonly cited support tool to increase adherence. The healthcare providers indicated that this strategy was successfully employed in TB and HIV programs, “*hav[ing] seen sending text message reminders work well with TB/HIV and believe it would work well for HT” (KII 6, physician).*

Lowering the price of HT medication (*n* = 6) and medication specific counseling (*n* = 2) were also mentioned by the healthcare providers, stating “*If it could be free to take medications, most patients would take it” (KII 5, physician), and “similar to TB/HIV, making medicines free would likely increase adherence” (KII 8, physician).*

Building effective partnerships by engaging religious leaders to inform patients about HT and the importance of taking medication was mentioned by four of the key informants. One male physician explained “*By using religious leaders to disseminate messages about lifestyle changes and the continuation of taking medicines, patients will hear the message and take it seriously…patients will understand that religion cannot cure their disease” (KII 3, physician).*

## Discussion

The findings from this study provide some preliminary insight on the barriers of patient medication adherence in primary care settings in Tanzania, including factors associated with provider training; quality and length of patient counseling on HT; patient understanding of medication adherence importance and consequences of non-adherence; and medication costs and healthcare access, which all play key roles. Many of the identified barriers by both patients and physicians were similar, including access, patient specific counseling, and patient reminders. These findings emphasize the need for multifaceted local solutions, as each barrier has the potential to reduce medication non-adherence for effective HT control.

### Patient level factors

Several studies have reported on barriers to adherence for antihypertensive medications from the patients’ perspective [16, 38, 39]. These include inconvenient clinic hours, long wait times, under-dispensing of prescriptions, side effects, faith-related behaviors, substitution of herbal supplements for pills, and lack of awareness of the need for continued medication use. In our study, affordability, patient knowledge and awareness, perception of feeling better or side effects, forgetfulness, and trust in the healthcare providers emerged as key patient related barriers.

Financial and access barriers including affordability of medication was mentioned in our study by both physicians and patients. Other studies have also reported similar findings on financial barriers, especially due to high out of pocket spending for poorer patients, [40, 41]. Free or subsidized medication, health insurance, and tax exemptions were mentioned in our study as a recommendation to improve treatment adherence. However, co-payment or free medication policies showed varied results for blood pressure control in the Nigerian studies [40, 41]. Policy level considerations may be required to address these options.

Similar to our study, where patients reported inadequate awareness of HT, 73.3% of the patients reported poor knowledge of HT in a study from Ghana. Counseling alone or counseling combined with patient training improved BP control in another study [42]. A systematic review of qualitative studies from 16 countries, including Tanzania [11, 43], showed that many patients intentionally reduced or stopped treatment without consulting their physicians. Patient-centric care and engaging patients in shared decision making have both been identified as important facilitators in addressing barriers to medication adherence [44].

Stopping medication when symptoms disappear, fear of addiction and side effects have been reported in other studies [11, 43]. Our study also showed that physicians felt that fear of side effects from long term medication use contributed to patient non-adherence to antihypertensive medications. Age, gender, place of residence, income, educational levels, number of prescriptions, price of medications, comorbidities, adverse effects, and not feeling the need for regular use have shown to be predictors of adherence to antihypertensive medications in studies conducted in Egypt, Pakistan, and Bangladesh [17, 18, 45]. Travel costs, long wait times, clinic operating hours, inadequate patient knowledge, and under-dispensing of medications were shown to be other limiting factors in a study in Nigeria [34], which were also mentioned by participants in our study.

Patient forgetfulness has been documented as a key contributor to non-adherence [2, 3], and provides both systems level and individual level interventions. A study from Ghana on patient-level factors reported that forgetfulness, along with medication side-effects and a high pill burden, were major factors for non-adherence to anti-HT medication [43]. Forgetfulness was also reported in our study, especially for older adults. In addressing patient centric care, the age of the patient is also an important consideration. Reminders from family members or the clinical team, use of pill boxes, special pill packaging, home or community-based distribution of refills, and home blood pressure monitoring may be feasible options to manage HT effectively [46]. Participants in the FGDs recommended linking daily medication to a routine task or habit.

Another important reported factor that influences patient medication adherence is trust in healthcare providers [2, 34]. The information from the FGD and KII in our study seemed to emphasize the importance of building and enhancing provider-patient trust, as patients tended to trust their religious leaders more than their physicians for authentic health information. Reinforcement of physician counseling by religious leaders would help medication adherence [34]. It has also been reported that patients who are accustomed to using alternative treatments to western medicine have lower anti-hypertensive medication adherence [47], also mentioned by healthcare providers in our study.

Improving patient education and counseling by providers and reinforcing messaging by religious leaders can be considered for immediate short-term remedies, before instituting long term strategies for pill boxes or organizers and enhanced access with community-based distribution.

### Physician and healthcare system related factors

The importance of complimenting behavioral medicine to pharmaceutical medicine for HT management has been proposed, advocating for investments at the patient level for education and empowerment, and at the physician level to address awareness of the cognitive and behavioral factors involved in patients’ active engagement for their care [48]. The lack of provider knowledge for appropriate patient counseling is also a key concern, as reported in our study and other studies in Tanzania [27, 49]. Additionally, mass public health messaging through the media does not reach those in the lowest socio-economic strata. Addressing these knowledge gaps has shown that when patients report moderate or good knowledge, there is an increased odds of HT control [50]. Efforts for an effective, patient centered, communication continuum may be needed to improve adherence to antihypertensive medication and lifestyle adherence, which begins with ensuring adequate knowledge competencies of healthcare providers.

Phone text messaging was proposed as a potential reminder system in our study, both by providers and patients. Some studies that focused exclusively on antiretroviral therapy showed increasing adherence to medication with SMS text messaging [51–53]. WhatsApp messaging has been shown to increase HT medication adherence by 15% in Brazil [54], and was mentioned as a strategy used by physicians to send patient reminders in our study. The use of SMS text reminders and WhatsApp can be used as reinforcement measures for improving adherence.

The medical team at the Community Center for Preventive Medicine center has launched numerous innovations, including household and community patient follow up, SMS text messaging, and partnering with health officials to expand programming aimed at preventing and screening for HT, including events conducted in the marketplace and at churches. These efforts must be supported and empirically evaluated as effective solutions for HT control, as patient education strategies have shown evidence of positive promotive behaviors [55].

Travel costs, long wait times, clinic operating hours, inadequate patient knowledge, and under-dispensing of medications were shown to be other limiting factors in a study in Nigeria [34], which were also mentioned by participants in our study. Predictors of antihypertensive medication include age, gender, place of residence, income, educational levels, number of prescriptions, price of medications, comorbidities, adverse effects, and not feeling the need for regular use [17, 18, 45].

Findings from our study indicate that access to affordable primary care including lower cost or free medications emerges as a key solution to improve adherence to hypertensive medication. The emphasis for patient reminders using SMS by the clinicians and family and community support factors for supporting patient adherence to antihypertensives is a key finding that emerged from this study, providing opportunities for future programming on effective HT control. The growing burden of HT prevalence in LMICs like Tanzania necessitates effective management with local solutions to improve access for affordable care and treatment. Given the serious health risks associated with uncontrolled or under-treated HT, including stroke, heart attack and chronic kidney disease, the issues of appropriate treatment and adherence are becoming increasingly critical.

The research was conducted in a specific semi-urban community in Tanzania, with unique contextual elements of patient characteristics, geographic and financial access, and health system factors, and therefore the transferability of the study may be limited. Future studies that are empirically designed to integrate the elements of the EMERGE guidelines are warranted to appreciate the complexity patient medication adherence and determine contextually relevant strategies for addressing the barriers [37].

## Conclusion

To achieve the global target of a 25% reduction in elevated blood pressure [56], contextually relevant multifaceted interventions are needed to ensure that patients diagnosed with HT have continuous lifetime hypertensive therapy to achieve optimal control [9]. This qualitative study provides evidence on factors that impede patient medication adherence, and potential strategies to effectively address these gaps in the patient care continuum, in semi-rural communities in Tanzania. While strategies focused on patient and provider factors should be implemented, policy level changes, ideally guided by rigorous intervention studies, are needed to improve the medication adherence of patients with HT.

## Supplementary Information

**Additional file 1.** Focus Group Discussion Guide for Patients with Hypertension.

## Data Availability

Data sharing is not applicable to this article as no datasets were generated or analyzed during the current study.

## Abbreviations

FGD: Focus Group Discussion
HT: Hypertension
KII: Key Informant Interview
SBP: Systolic Blood Pressure
DBP: Diastolic Blood Pressure
