# Supplementary material for: Patient and healthcare provider perspectives on adherence with antihypertensive medications: an exploratory qualitative study in Tanzania
Source: BMC Health Serv Res. 2021 Aug 18;21:834. doi: 10.1186/s12913-021-06858-7 (PMC8371775; doi:10.1186/s12913-021-06858-7)
Supplement: Supplementary file 1 — Additional file 1. Focus Group Discussion Guide for Patients with Hypertension. [file 12913_2021_6858_MOESM1_ESM.docx]

BMC Health Services Research Manuscript Supplemental File

Title: Patient and Healthcare Provider Perspectives on Adherence with Antihypertensive Medications: A Qualitative Study in Tanzania

Authors: Anbrasi Edward, Brady Campbell, Frank Manase, Lawrence J Appel

***Focus Group Discussion Guide for Patients with Hypertension***

**1. Current Understanding of hypertension [PROBE ]**

- Do you think people in the community have heard of high blood pressure or hypertension (HTN)?
- Do you know anyone in your family or community with HTN?
  - Lots of people, older people, pregnant women, obese people etc
- Are there any HTN screening programs in this community?
- When people in this community fall sick and visit the health center do they take their BP?
- Can we treat high BP?
- What are the ways we can reduce high BP?
  - Lifestyle modification like not using or reducing smoking and alcohol, increasing physical activity and exercise, increasing fruits and vegetables in diet, reduce salt in cooking or adding to food, reducing foods high in sodium like packaged foods, preservatives, pickles etc
- Why do people believe we need to treat HTN? how can it be treated? in what ways?

**2. Understanding the role of medications [PROBE ]**

- Are you aware of family or community members who take medication for HTN?
- When is it appropriate not to take HTN medication?
- Should people take HTN medication even if they are feeling well? If yes, explain why.
- How often do people with HTN have to take medications?

**3. Reasons for not taking medication [PROBE ]**

- Do you know people in the family or community who might not take their medication regularly every day?
- What are the reasons for not taking their HTN medication?
- What barriers exist to taking HTN medications regularly?
  - Forget, inadequate supplies, clinic too far, medications expensive, feel better, access traditional healers, take other homeopath meds, control by diet and exercise
- If they obtain HTN medicine from health center, is it free, subsidized, how much do they pay, how often do they go to the clinic to get meds, is it easy to travel to the clinic, are HTN medications always available?
- If purchased from pharmacy, how expensive are they? Do they purchase full dose, or partial dose?

**4. Use of traditional medicine & Lifestyle changes [PROBE ]**

- Do you know if family or community members take traditional medicines for HTN?
- What type of traditional medicine do they take?
  - Define term if uncertain
- Is this effective in treating high BP?
- Why do people prefer to take traditional medicine?
- Are there things other than medications that can help with HTN?
- Have you seen or ever made lifestyle changes to help with HTN?
  - Diet
  - Alcohol
  - Exercise
  - Smoking

**5. Potential Solutions [PROBE ]**

- Could you tell us about some of the ways in which those on HTN medications remember to take medications?
  - Reminded by family member, keep medications near drinking water, pick a certain time, waking up, going to bed, bubble pack, text message etc.
- Would timing factors help adherence?
  - Taking it at the same time every day.
  - Taking it with meals/before bed/waking up? etc.
- Have you seen people trying to solve this problem before?
  - What has been tried?
- Have you seen people receive text messages in their cell phone to remind them to take HTN medications?
- Do you think text message is a good way of reminding people to take medications? Will this work in your family or community? Why? Why not?
- What are other ways you would suggest helping those on HTN medication take their medications regularly?
- Is reducing cost a potential solution?
  - free medication to help increase adherence?
  - subsidized medications to make them cheaper?
- Do people normally purchase medications at the clinic or at a pharmacy?
  - Would it be beneficial to have medications at the clinic?

1. **Physician Counseling [PROBE]**

- When people are diagnosed with high BP do they explain about the importance of taking medicines regularly?
- Do they explain about follow up?
- Do they talk about lifestyle modification like not using or reducing smoking and alcohol, increasing physical activity, increasing fruits and vegetables in diet?
- Do they inform about reducing sodium in the food, when cooking or at the table?
- Do you think physicians can provide better explanation about HTN?
  - If yes, what information would be useful
  - Do you feel like your physicians should provide you with more resources? if so, what?
- If we can change one thing to help people take medicine regularly, what would it be?

Key Informant Interview Guide for Primary Healthcare Providers Who Screen and Manage Patients with Hypertension

**1. Current Understanding of hypertension**

- Do you know what hypertension (HTN) or high blood pressure is?
- Do you feel comfortable treating HTN?
- Do you feel like you have received sufficient training to treat HTN? If no, explain.
  - Is there a resource that could be provided to you to help you better understand HTN?
- What are some long-term consequences of HTN?

**2. Hypertension in clinic**

- How often do you see HTN in clinic?
- What do you think patients understand about HTN?
- Do you think patients take their medicine?
- Do you counsel patients in clinic? What types of messages do you provide, taking meds regularly, follow up, diet, exercise, reduce/eliminate smoking, alcohol, reduce dietary salt and high sodium foods
  - If so, do you think this helps them with adherence?
- What patients do you find them most difficult to counsel?
  - Young vs old, Male vs Female, SES, jobs, education level etc.

**3. Use of traditional healers and lifestyle medicine**

- Do you think that patients seek care from traditional healers? If so, why?
- How many patients seek care from traditional healers?
- What are benefits of seeking care from traditional healers?
- Do you believe that cost is a determinant in seeking care from traditional healers?
- Are there lifestyle changes patients can make to help with HTN?
  - Do you counsel your patients about lifestyle changes?
    - Diet, Sodium
    - Smoking
    - Stress
    - Exercise
    - Alcohol

**4. Barriers to Medication**

- When you prescribe medications, how many pills do patients take per day?
  - How many times per day do patients take pills?
- What do patients have to do in order to get and take their medicine every day?
  - Do you provide medications at your clinic?
  - How far do people have to travel to get medications?
- How long can you write a prescription for?
- Do patients ever say that cost is a barrier?
  - How much do medications typically cost people?
  - Are there programs where patients can get medications for free?
  - Any programs to subsidize medications?
- Within that process, what are some places you think could be potential problems?
  - If they do not list any, ask about price, access, stigma, remembering to take it, etc.
- Which barrier do you think is the biggest problem?
- Are there any barriers you feel are more present in your community than others?
- What do you think patients say their main barrier?

**5. Solutions**

- What methods have you seen to be successful for patients who adhere to medications?
- What do you think is the most successful intervention that has been used to increase adherence?
- Do you have any ideas about potential interventions that could increase adherence?
- Potential solutions?
  - SMS reminders
  - Patient education materials
  - Direct observation
  - CHW
  - Family reminders
  - Calendar
  - Pillbox
  - Setting pills beside water
- Is there a way that physicians could partner with traditional medicine to increase adherence?
- Is there a tool that could help you better educate your patients?
